# The Heme Oxygenase-1 Inducer THI-56 Negatively Regulates iNOS Expression and HMGB1 Release in LPS-Activated RAW 264.7 Cells and CLP-Induced Septic Mice

**DOI:** 10.1371/journal.pone.0076293

**Published:** 2013-10-03

**Authors:** Eun Jung Park, Hwa Jin Jang, Konstantin Tsoyi, Young Min Kim, Sang Won Park, Hye Jung Kim, Jae Heun Lee, Ki Churl Chang

**Affiliations:** Department of Pharmacology, School of Medicine Gyeongsang National University and Institute of Health Sciences, Jinju, Korea; University of California Los Angeles, United States of America

## Abstract

The nuclear DNA binding protein high mobility group box 1 (HMGB1) has recently been suggested to act as a late mediator of septic shock. The effect of ((S)-6,7-dihydroxy-1-(4-hydroxynaphthylmethyl)-1,2,3,4-tetrahydroisoquinoline alkaloid, also known as THI-56, in an experimental model of sepsis was investigated. THI-56 exhibited potent anti-inflammatory properties in response to LPS in RAW 264.7 cells. In particular, THI-56 significantly inhibited the expression of inducible nitric oxide synthase (iNOS) and the release of HMGB1 in activated macrophages. THI-56 activated NE-F2-regulated factor 2 (Nrf-2)/heme oxygenase 1 (HO-1). The specific knockdown of the HO-1 gene by HO-1 siRNA significantly reversed the inhibitory effects of THI-56 on iNOS expression and HMGB1 release in LPS-stimulated macrophages. Importantly, THI-56 administration protected animals from death induced by either a lethal dose of LPS or cecal ligation and puncture (CLP). Furthermore, the ALT, AST, BUN, creatinine, and HMGB1 levels in the blood were significantly increased in CLP-induced septic mice, and the administration of THI-56 reduced these levels in a concentration-dependent and zinc protoporphyrin IX (ZnPPIX)-sensitive manner. In addition, the administration of THI-56 significantly ameliorated not only lung damage but also macrophage infiltration in the livers of CLP-induced septic mice, and these effects were also abrogated in the presence of ZnPPIX. Thus, we conclude that THI-56 significantly attenuates the proinflammatory response induced by LPS and reduces organ damage in a CLP-induced sepsis model through the upregulation of Nrf-2/HO-1.

## Introduction

Sepsis is defined as a systemic inflammatory response syndrome resulting from a microbial infection. Despite recent advances in antibiotic therapy and intensive care, sepsis is still the most common cause of death in intensive care units [1]. The pathogenesis of sepsis is attributable, at least in part, to dysregulated systemic inflammatory responses characterized by the excessive accumulation of various proinflammatory mediators, such as tumor necrosis factor (TNF) or interleukin (IL)-1 [2], interferon-gamma (IFN-γ) [3], and nitric oxide (NO) [4]. Recently, it has been demonstrated that a ubiquitous protein, high mobility group box 1 (HMGB1), is released by activated macrophages/monocytes and functions as a late mediator of lethal endotoxemia and sepsis [5]–[8].

Heme oxygenase-1 (HO-1), a stress-responsive protein that can be induced by stimulants such as inflammatory cytokines, heat shock, heavy metals and oxidants, degrades heme into three products: Fe^++^, biliverdin, and carbon monoxide (CO). Biliverdin is subsequently converted into bilirubin by biliverdin reductase. The increased level of Fe^2+^ stimulates the synthesis of ferritin (an iron-binding compound), which is known to be a cytoprotective antioxidant protein. The HO-1 system has anti-apoptotic, anti-oxidant and immunomodulatory functions under various conditions [9]–[11]. Much attention has been paid to CO and CO-releasing molecules (CORMs) due to their ability to attenuate inflammatory responses in many different experimental models [12]–[17]. Recently, we and others demonstrated that the HO-1/CO system can play a very important role in sepsis through the negative regulation of HMGB1 [18]–[19]. Thus, it seems plausible that the identification of HO-1-inducing agents can lead to the development of therapeutic interventions for inflammatory disorders such as sepsis.

**Figure 1:**
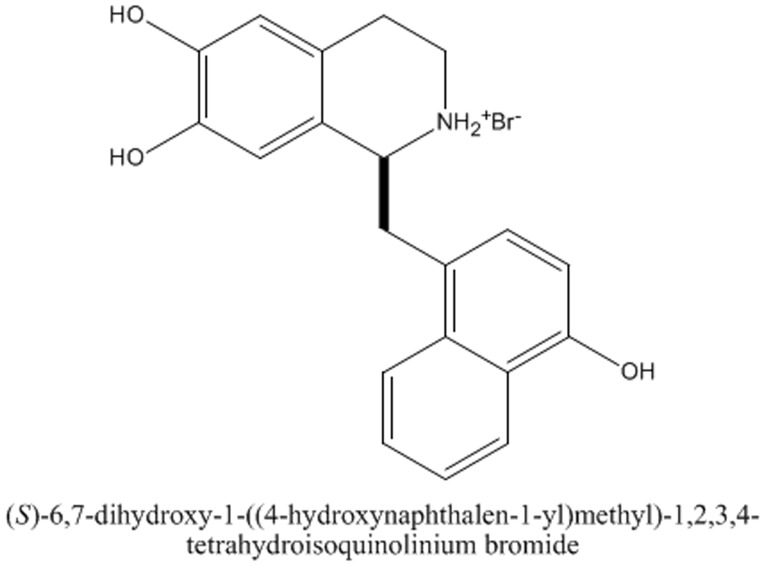
Chemical structure of THI-56.

Tetrahydroisoquinoline (THI) alkaloids are of special interest due to their pharmacological effects on inflammation and related disorders [20]–[27]. Previously, we reported that THI alkaloids can induce HO-1 expression in many cells, including RAW 264.7 cells [20], [25]–[26]. Herein, we report that a novel compound, (S)-6,7-dihydroxy-1-(4-hydroxynaphthylmethyl)-1,2,3,4-tetrahydroisoquinoline (THI-56, Fig. 1), induces HO-1 protein expression in RAW 264.7 cells and in the tissues of cecal ligation and puncture (CLP)-induced septic mice. This increased HO-1 expression significantly attenuated the proinflammatory response and reduced the level of circulating HMGB1.

## Materials and Methods

### Materials

The anti-HMGB1 antibody was purchased from Abcam (Cambridge, MA), and the anti-iNOS antibody was obtained from Transduction Laboratories (Lexington, KY). The anti-β-actin, anti-Nrf-2 and anti-heme oxygenase-1 antibodies were supplied by Santa Cruz Biotechnology (Santa Cruz, CA). The enhanced chemiluminescence (ECL) western blotting detection reagent was purchased from Amersham (Buckinghamshire, UK). All other chemicals, including, LPS (*Escherichia coli* O111:B4) and zinc protoporphyrin IX (ZnPPIX), were purchased from Sigma-Aldrich (St. Louis, MO). The HMGB1 detection kit was purchased from Shino Test Corp. (Tokyo, Japan). The alanine aminotransferase (ALT), aspartate aminotransferase (AST), and blood urea nitrogen (BUN) assay kits were obtained from IVD Lab Corp. (Uiwang, Korea). The enzymatic creatinine reagent kit was purchased from Sekisui Medical (Tokyo, Japan).

### Cell Culture and Stimulation

RAW 264.7 cells were obtained from the American Type Culture Collection (ATCC, Rockville, MD). The cells were grown in RPMI-1640 medium supplemented with 25 mM N-(2-hydroxyethyl)piperazine-N-2-ethanesulfonic acid (HEPES), 100 U/ml penicillin, 100 µg/ml streptomycin and 10% heat-inactivated fetal calf serum. RAW 264.7 cells were plated at a density of 1×10^7^ cells per 100 mm dish. The cells were rinsed with fresh medium and stimulated with LPS (1 µg/ml) in the presence or absence of different concentrations of THI-56 (1, 10 and 25 µM). THI-56 was dissolved in sterile distilled water, and the solution was sterilized using a 0.2 µm filter.

### Cell Viability

Cell viability was assessed colorimetrically using the MTT assay. Cells in the exponential phase were seeded at 1×10^4^ cells/well in 24-well plates. After different treatments, 20 µl of 5 mg/ml MTT solution was added to each well (0.1 mg/well), and the plates were incubated for 4 h. The supernatants were aspirated, the formazan crystals in each well were dissolved in 200 µl of dimethyl sulfoxide for 30 min at 37°C, and the optical density at 570 nm was read on a microplate reader (Bio-Rad, Hercules, CA).

### Assay for NOx Production

The NO level was determined by measuring the level of its stable oxidative metabolite, nitrite (NOx), with Griess reagent as described previously [21]. At the end of the incubation, 100 µl of the culture medium was mixed with an equal volume of Griess reagent (0.1% naphthylethylenediamine dihydrochloride and 1% sulfanilamide in 5% phosphoric acid). The absorbance at 550 nm was measured, and the nitrite concentration was determined using a calibration curve constructed with sodium nitrite standards.

### Western Blot Analysis

The cytoplasmic/nuclear fractionation was performed using a nuclear/cytosol fractionation kit (Cat # K266-25, BioVision, Mountain view, CA) according to the manufacturer’s manual. Total protein was obtained using lysis buffer containing 0.5% SDS, 1% Nonidet P-40, 1% sodium deoxycholate, 150 mM NaCl, 50 mM Tris-Cl (pH 7.5), and protease inhibitors. The protein concentration of each sample was determined using a BCA protein assay kit (Pierce, Rockford, IL). To detect iNOS and HO-1, 20 µg aliquots of the total protein were electrophoresed on 8% and 10% polyacrylamide gels, respectively. The electrophoresed proteins were transferred to polyvinylidene difluoride (PVDF) membranes by semidry electrophoretic transfer at 15 V for 60–75 min. The PVDF membranes were blocked overnight at 4°C in 5% bovine serum albumin (BSA). The membranes were incubated with primary antibodies diluted 1∶500 in Tris-buffered saline/Tween 20 (TBS-T) containing 5% BSA for 2 h and then incubated with the secondary antibody at room temperature for 1 h. Anti-rabbit IgG was used as the secondary antibody (1∶5000 dilution in TBST containing 1% BSA). The signals were detected by ECL (Amersham, Piscataway, NJ).

### siRNA Technique

HO-1 small interfering RNA (siRNA) was purchased from Invitrogen, and siNrf-2 was purchased from Santa Cruz Biotechnology (Santa Cruz, CA). The sequence of the mouse HO-1 siRNA (5′ to 3′) was as follows: (RNA) – UUA CAU GGC AUA AAU UCC CAC UGC C. The siRNAs were transfected into RAW 264.7 cells according to the manufacturer’s protocol using the SuperFect transfection reagent from QIAGEN. The cells were incubated with the HO-1 and Nrf-2 siRNAs at 100 nM for 16 h in serum-free media. After incubation, the transfected cells were subjected to treatment as described in the figure legends.

### HMGB1 Analysis

HMGB1 was analyzed as described previously [19]. Culture medium samples were briefly centrifuged to remove cellular debris. Equal volumes of samples were then concentrated 40-fold with Amicon Ultra-4-10000 NMWL filter units (Millipore). The centrifugation conditions were a fixed angle (35 degree) and 7500 g for 20 min at 4°C. Then, the concentrated samples were mixed with 2x loading dye and boiled at 95°C for 5 min. The proteins were separated on 12% SDS-polyacrylamide gels and transferred to immunoblot membranes. The membranes were blocked with 5% BSA overnight at 4°C. After blocking, the membranes were washed with Tris-buffered saline/Tween 20 (TBS-T) buffer for 1 h at room temperature. Next, the membranes were incubated with an anti-HMGB1 antibody (Abcam, 1∶1000) at 4°C for 16 h. After incubation, the membranes were washed with TBS-T for 1 h at RT and incubated with a goat anti-rabbit IgG-HRP secondary antibody (1∶5000 dilution in TBST containing 1% BSA). The signals were detected by ECL (Amersham, Piscataway, NJ).

### HO-1 Activity

Measurment of HO-1 activity was done as previously reported [28]. In brief, the harvested cells were conduced to three times of freeze-thawing and then addition to a reaction mixture consisting of phosphate buffer (1 ml final volume, pH 7.4) containing MgCl_2_ (2 mM), glucose-6-phosphate (2 mM), glucose-6-phosphate dehydrogenease (0.2 units), NADPH (0.8 mM), the substrate hemin (20 µM), and rat liver cytosol as a source of biliverdin reductase. The reaction mixture was incubated at 37°C for 1 h and completed by the addition of 1 ml of chloroform. After vortexed and centrifuged, the bilirubin of chloroform layer was examined by the contrast in absorbance between 464 and 530 nm (*ε* = 40 mM^−1^ cm^−1^).

### Ethics Statement

Mice were maintained in accordance with the Guide for the Care and Use of Laboratory Animals (NIH publication 85-23, revised 1996) and were treated ethically. The protocol was approved in advance by the Animal Research Committee of the Gyeongsang National University.

### Animal Model of Endotoxemia and CLP-induced Sepsis

Endotoxemia was induced in BALB/c mice (male, 7–8 wk, 20–25 g, Koatech, Korea) by the i.p. injection of bacterial endotoxin (LPS, 15 mg/kg). To induce CLP-induced sepsis, BALB/c mice were anesthetized with ketamine (30 mg/kg) and xylazine (6 mg/kg). Next, a 2-cm midline incision was made to allow exposure of the cecum with the adjoining intestine. The cecum was tightly ligated with a 3.0-silk suture 5.0 mm from the cecal tip and punctured once with a 22-gauge needle. The cecum was then gently squeezed to extrude a small amount of feces from the perforation site and returned to the peritoneal cavity. The laparotomy site was then closed with 4.0 silk. In sham control animals, the cecum was exposed but not ligated or punctured and then returned to the abdominal cavity.

### Histological Examination

Twenty-four hours after CLP, all animals were sacrificed under ketamine (30 mg/kg, i.p.) anesthesia. The kidneys and the superior lobe of the right lung were excised for histopathological examination, fixed with 4% paraformaldehyde in PBS and embedded in paraffin wax. Four-micron-thick sections were cut using a microtome, and the deparaffinized tissue sections were subjected to hematoxylin and eosin (H&E) staining for histological examination. The lung injury scores were measured in section using neutrophil infiltration, hemorrhage, necrosis, congestion and edema [29]. Each injury was scored according to the following system (0 =  normal; 1≤25%; 2 = 25–50%; 3 = 50–75%; 4≥75%). The sections were conducted by an investigator blinded to the treatment. To assess the infiltration of macrophages in the liver, deparaffinized liver sections were placed in a solution of 1% H_2_O_2_ for 10 min. After washing, the sections were incubated in 5% blocking serum for 1 h at room temperature. The slides were incubated overnight at 4°C in a humidified chamber with anti-rabbit-F4/80 (1∶50; Santa Cruz Biotechnology). After being washed three times with 0.1 M PBS, the sections were incubated for 1 h at room temperature with a secondary biotinylated antibody (1∶100). After washing, the sections were incubated in an avidin-biotin-peroxidase complex solution (Vector Laboratories, Burlingame, CA). The sections were developed with 0.05% diaminobenzidine (Sigma-Aldrich) containing 0.05% H_2_O_2_, dehydrated, and coverslipped with Permount (Sigma-Aldrich). The sections were visualized under a CKX41 light microscope (Olympus).

### Statistical Evaluation

The data are expressed as the mean ± SEM of the results obtained from the specified number of replicate treatments. Differences between data sets were assessed by one-way analysis of variance followed by the Newman-Keuls tests. The Kaplan-Meier method was used to compare the differences in the mortality rates between groups. P<0.05 and P<0.01 were accepted as statistically significant.

## Results

### THI-56 Significantly Attenuated iNOS/NO and HMGB1 Release in Macrophages Induced by LPS

**Figure 2:**
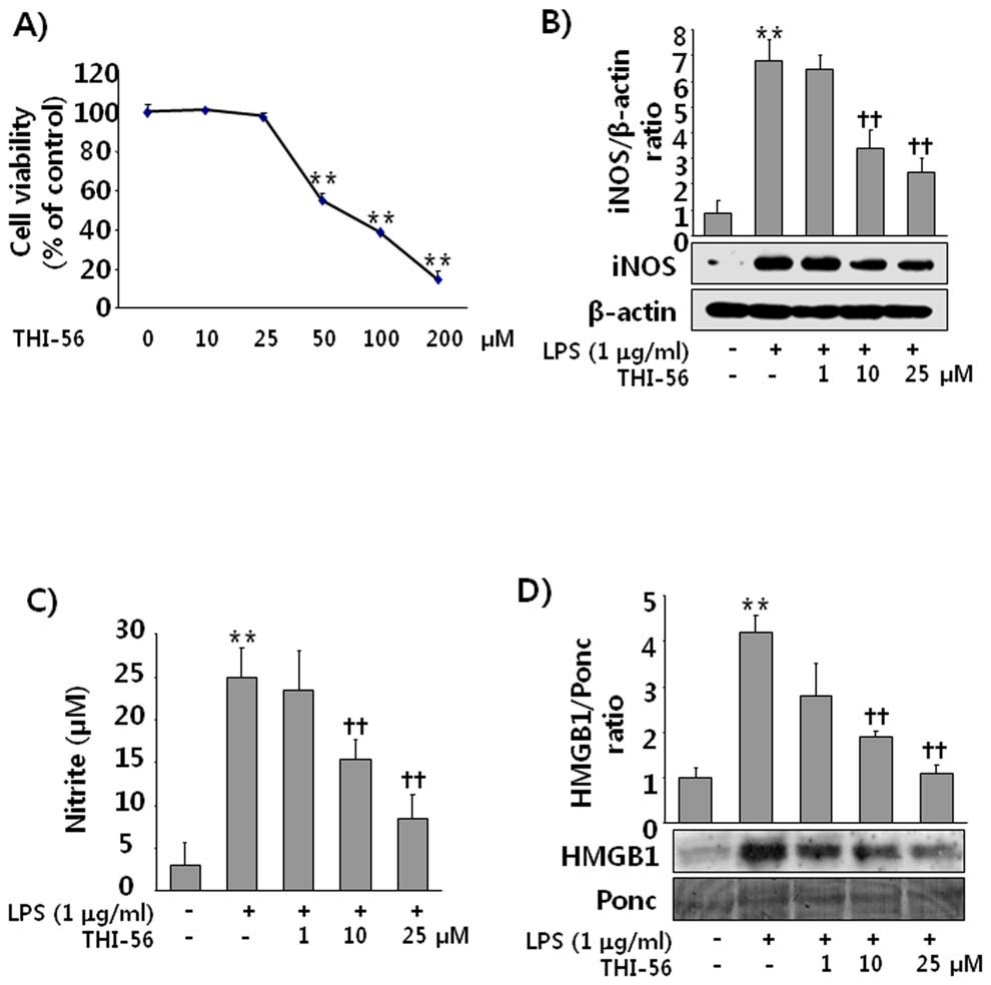
Effect of THI-56 on the expression levels of iNOS/NO and HMGB1 in LPS-stimulated macrophages. Cells were treated with THI-56 (10, 25, 50, 100 and 200 µM) for 24 h, and then the MTT assay (A) was performed as described in the Materials and Methods. Cells were treated with LPS (1 µg/ml) with and without THI-56 (1, 10, 25 µM) for 16 h. After incubation, the cells were lysed for iNOS detection (B). Culture medium was also collected for NO measurements (C) and HMGB1 analysis (D) as described in the Materials and Methods. The data are presented as the means ± SEM of three independent experiments. One-way analysis of variance followed by the Newman-Keuls test was used to compare the means of multiple groups (significant compared with the control, **P < 0.01; significant compared with LPS,^ ††^ *P* < 0.01).

We first assessed the effect of THI-56 on cell viability. Figure 2A shows that there was a significant loss of cell viability at concentrations of 50 µM and above, whereas no effect was found on cell viability up to 25 µM. Thus, for the subsequent experiments, we used concentrations less than 50 µM. We next were interested whether THI-56 can affect the proinflammatory responses of activated macrophages. As shown in Fig. 2B, C, and D, THI-56 significantly inhibited the production of iNOS/NO and the release of HMGB1 induced by LPS, suggesting that THI-56 has potent anti-inflammatory effects on LPS-activated macrophages.

### THI-56 Induces HO-1 Expression Through the Nrf-2 Transcription Factor in Macrophages

**Figure 3:**
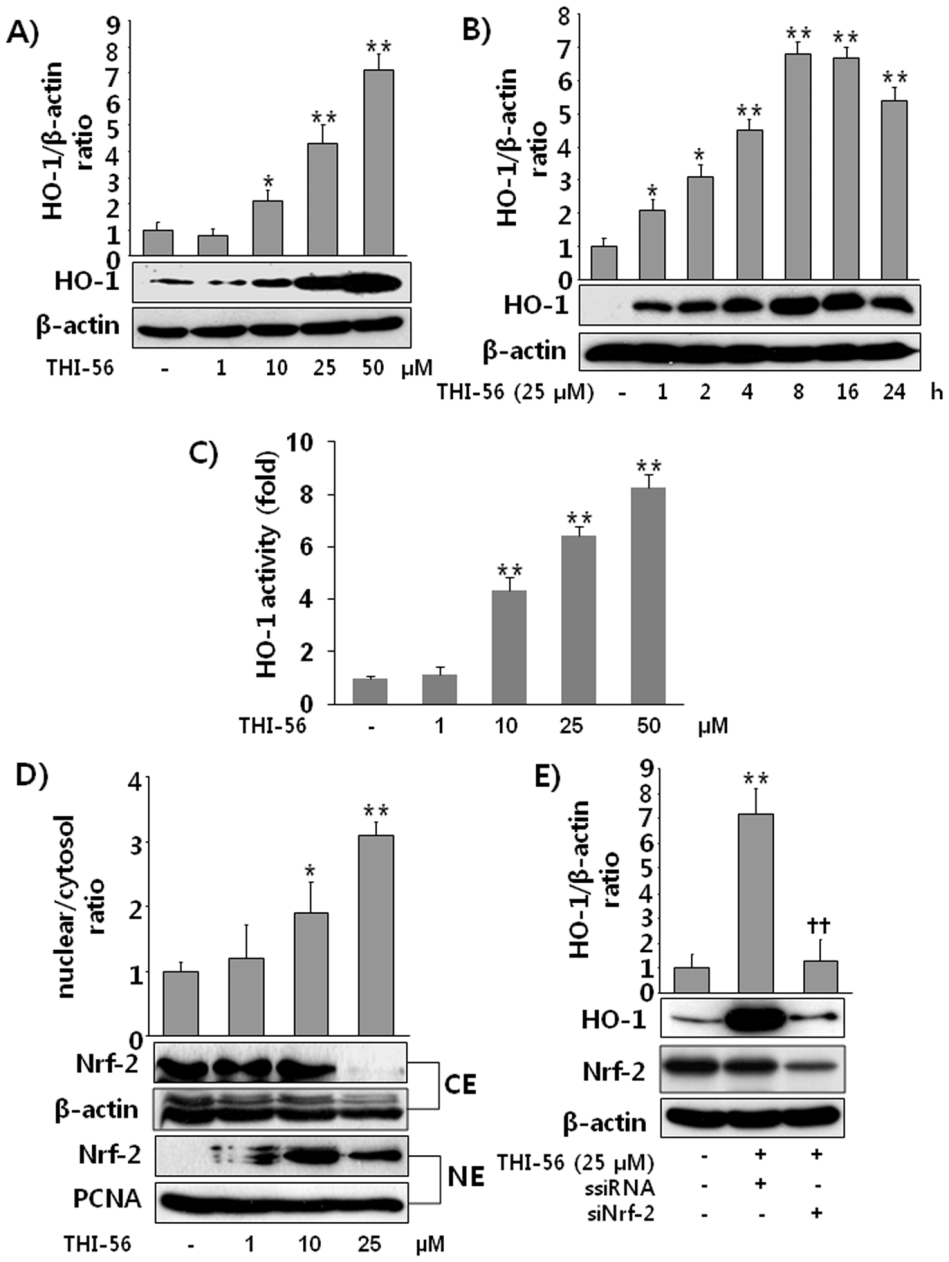
Effect of THI-56 on HO-1 expression and Nrf-2 activation in macrophages. Cells were treated with THI-56 (1, 10, 25, or 50 µM) for 8 h (A) or with THI-56 (25 µM) for 1, 2, 4, 8, 16, or 24 h. (B). After incubation, the cells were lysed for western blot analysis and HO-1 activity (C) was measured as described in the Materials and Methods. Cells were treated with THI-56 (1, 10, or 25 µM) for 8 h. After incubation, cytosol/nuclear fractionation was performed using a cytosol/nuclear fractionation kit, and samples were subjected to western blot analysis (D). Cells were transfected with scramble siRNA (ssiRNA) (100 nM) or siNrf-2 (100 nM) and incubated for 16 h. After incubation, the transfected cells were treated with THI-56 for 8 hand the cells were lysed and subjected to western blot analysis (E). The data are presented as the means ± SEM of three independent experiments. One-way analysis of variance followed by the Newman-Keuls test was used to compare the means of multiple groups (significant compared with the control, *P < 0.05 **P < 0.01; significant compared with ssiRNA,^ ††^ *P* < 0.01).

We next asked whether THI-56 can upregulate the expression of the HO-1 protein. It was clearly demonstrated that HO-1 expression and HO-1 activity is significantly induced by THI-56 treatment in a concentration- and time-dependent manner in macrophages (Fig. 3A, B, and C). Most of the genes encoding phase II detoxifying and antioxidant enzymes have an antioxidant redox element (ARE) sequence in their promoter regions. NE-F2-regulated factor 2 (Nrf-2) is an important transcription factor that regulates ARE-driven HO-1 gene expression [30]. We therefore asked whether the HO-1-inducing effect of THI-56 is mediated by Nrf-2. THI-56 induced the translocation of Nrf-2 from the cytosol into the nucleus (Fig. 3D), and the knockdown of Nrf-2 using a specific siRNA abolished the HO-1 expression induced by THI-56 (Fig. 3E). According to these data, we can conclude that THI-56 induces HO-1 expression through Nrf-2 activation.

### HO-1 siRNA Abrogated the Anti-inflammatory Effect of THI-56 on LPS-activated Macrophages

**Figure 4:**
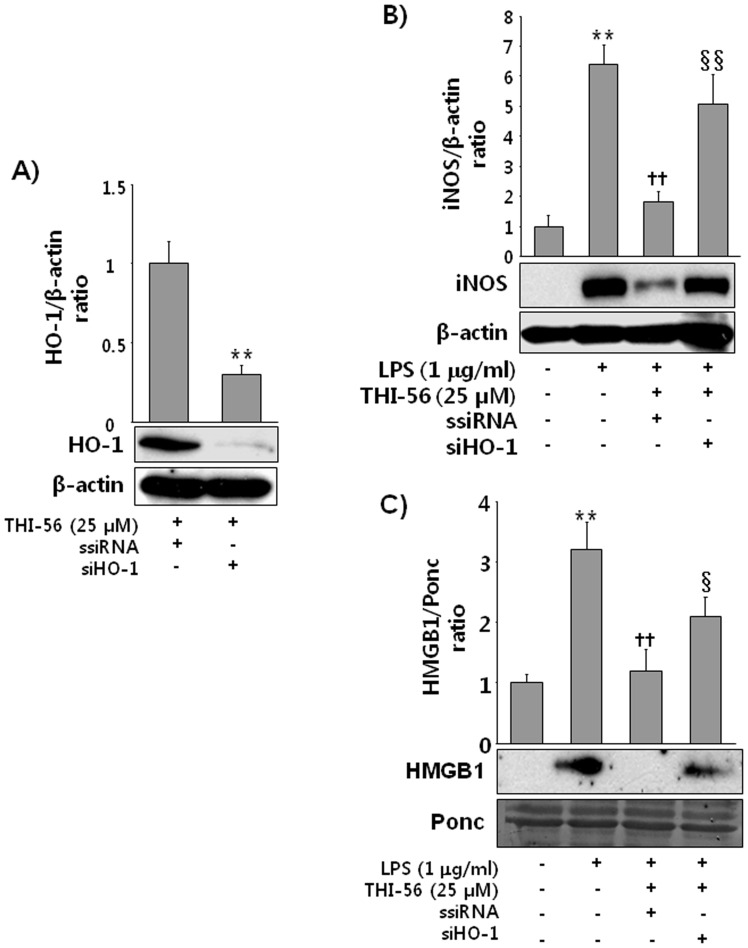
The anti-inflammatory effect of THI-56 was reversed in HO-1-knockdown cells. Cells were transfected with ssiRNA or siHO-1 and transfection efficiency was checked (A). Then, the cells were treated with THI-56 (25 µM) for 8 h. After incubation, the cells were lysed, and western blot analysis was performed. Cells were transfected with ssiRNA or siHO-1 as described above. Next, cells were stimulated with LPS (1 µg/ml) for 16 h in the presence or absence of THI-56 (25 µM). After incubation, the cells were lysed for iNOS detection (B), and culture medium was collected for HMGB1 analysis (C). The data are presented as the means ± SEM of three independent experiments. One-way analysis of variance followed by the Newman-Keuls test was used to compare the means of multiple groups (significant compared with the control, **P < 0.01; significant compared with LPS,^ ††^ *P* < 0.01, significant compared with ssiRNA, ^§^ *P*<0.05, ^§§^ *P*<0.01).

Because THI-56 significantly upregulated HO-1 expression in macrophages, we were interested in whether the HO-1-induction effect has an important role in the THI-56-mediated anti-inflammatory effect. As shown in Fig. 4A, transfection efficiency of siHO-1 was more than 80%. THI-56 significantly inhibited the LPS-induced increases in the iNOS (Fig. 4B) and HMGB1 (Fig. 4C) levels in scramble siRNA-transfected cells. However, THI-56 failed to do elicit this response in siHO-1-transfected cells, suggesting that the beneficial anti-inflammatory effects of THI-56 are mediated through HO-1 induction.

### THI-56 Improves Survival in Experimental Sepsis Models Through HO-1

**Figure 5:**
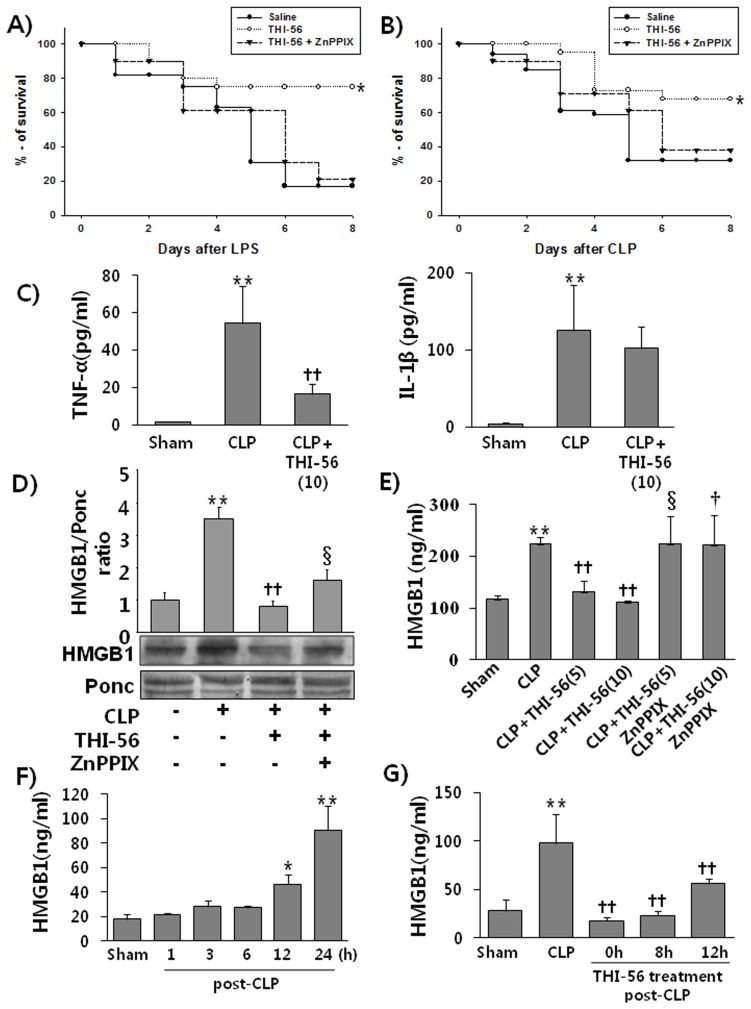
Effect of THI-56 on animal mortality induced by lethal endotoxemia and CLP. THI-56 (10 mg/kg, i.p., n = 20), THI-56+ZnPPIX (5 mg/kg, i.p., n = 20) or saline (i.p., n = 20) was administered 2 h prior to LPS administration or CLP. Then, the mice were subjected to lethal endotoxemia (LPS, 15 mg/kg, i.p.) (A) or CLP (B). Survival was monitored daily for up to 10 days. In separate experiments, BALB/c mice were treated with THI-56 (10 mg/kg, i.p., n = 6), THI-56+ZnPPIX (5 mg/kg, i.p., n = 8) or saline (i.p., n = 3) 2 h prior to CLP. Six hour after CLP, blood was taken from cardiac puncture under anesthesia. The blood TNF-α and IL-1β levels were measured (C). For measurement of HMGB1, BALB/c mice were treated with THI-56 (5 mg/kg, i.p., n = 5), THI-56 (10 mg/kg, i.p., n = 5), THI-56 (10 mg/kg, i.p)+ZnPPIX (5 mg/kg, i.p., n = 5) or saline (i.p., n = 3) 2 h prior to CLP. At 24 h after CLP, blood was collected by cardiac puncture under anesthesia, and HMGB1 analysis (D,E) was performed. Finally, we were curious whether post treatment of THI-56 was still reduce circulating HMGB1 in CLP mice, we firstly checked changes of HMGB1 levels according to the times after CLP. The blood HMGB1 levels were measured at 1, 3, 6, 12, and 24 h after CLP (n = 9) (F). To determine the effect of THI-56 on the blood HMGB1 level after CLP, THI-56 (10 mg/kg, i.p, n = 8) was administered at 0 h, 8 h, and 12 h after CLP. At 24 h after CLP, blood was collected by cardiac puncture under anesthesia, and HMGB1 analysis (G) was performed. The data are presented as the means ± SEM. One-way analysis of variance followed by the Newman-Keuls test was used to compare the means of multiple groups (significant compared with the control, * P<0.05, **P < 0.01; significant compared with CLP,^ ††^ *P* < 0.01, significant compared with, THI-56 alone, ^†^,§*P*<0.05). The Kaplan-Meier method was utilized to compare the differences in the mortality rates between groups. Significant compared with saline, *P <0.05.

In light of the ability of THI-56 to attenuate LPS-induced HMGB1 release, we explored its efficacy in an animal model of lethal endotoxemia. The administration of THI-56 at 10 mg/kg significantly improved survival; however, the protective effect THI-56 was completely abrogated by co-treatment with ZnPPIX (an HO-1 inhibitor) (Fig. 5A). Although endotoxemia is a useful animal model to investigate complex cytokine cascades, a more clinically relevant animal model for sepsis should also be used. One well-characterized, standardized animal model of sepsis is the CLP model. It has been shown that the administration of anti-HMGB1 rescues animals from septic death in this model [8]. We found that within a few hours after CLP, the animals demonstrated overt signs of illness, including piloerection, hunched posture, and diarrhea. Although these features were also shown in some mice of lower dose (5 mg/kg) of THI-56-treated group, the magnitude and frequency of these characters were much less in higher dose (10 mg/kg) of THI-56-treated mice. We therefore asked whether THI-56 can affect the death rate in this model. As expected, the administration of THI-56 significantly improved survival in the CLP-induced sepsis model (Fig. 5B). This increase in survival caused by THI-56 may be related to reductions of the levels of circulating proinflammatory cytokines, such as TNF-α and IL-1β. When the TNF-α and IL-1β levels were measured 6 h after CLP, increases in the levels of TNF-α and IL-1β of approximately 8-fold and 31-fold, respectively, compared with the levels in the sham animals were observed. However, treatment with THI-56 (10 mg/kg, i.p.) 2 h prior to CLP significantly reduced the circulating TNF-α levels (Fig. 5C). The level of IL-1β tended to decrease with THI-56 administration, but this change was not statistically significant (Fig. 5C). Not only TNF-α but also HMGB1 plays a crucial role in lethality during sepsis. To further confirm that the increased survival induced by THI-56 is associated with both the blood HMGB1 levels and HO-1-dependent phenomena, the serum HMGB1 levels in CLP-mice were compared in the presence and absence of either THI-56 or ZnPPIX. The elevated serum HMGB1 level in the CLP group was significantly reduced by THI-56 according to the western blot analysis. However, THI-56 failed to reduce the serum HMGB1 level in ZnPPIX-treated mice (Fig. 5D). More clear results were obtained for the circulating concentration of HMGB1. As shown in Fig. 5E, the circulating HMGB1 levels were clearly reduced by the administration of THI-56 to CLP-induced septic mice in a concentration-dependent manner, and this reduction was inhibited by ZnPPIX. In addition, we measured the changes in the blood levels of HMGB1 after CLP. As shown in Fig. 5F, the HMGB1 concentration started to increase after 12 h and continued to rise until 24 h after CLP. We therefore were curious whether the blood HMGB1 levels can be reduced by THI-56 when administered after CLP. Mice were subjected to CLP and then treated with THI-56 (10 mg/kg, i.p) at 0, 8, and 12 h after CLP. At 24 h after the CLP operation, blood was collected by cardiac puncture from ketamine-anesthetized (30 mg/kg, i.m.) mice. Fig. 5G clearly shows that the administration of THI-56 after CLP significantly reduced the circulating HMGB1 level. Even when given 12 h after CLP, THI-56 still significantly reduced the blood HMGB1 levels in CLP septic mice.

### THI-56 Protects Against Multiple Organ Failure in Sepsis Models in a Dose-dependent Manner

**Figure 6:**
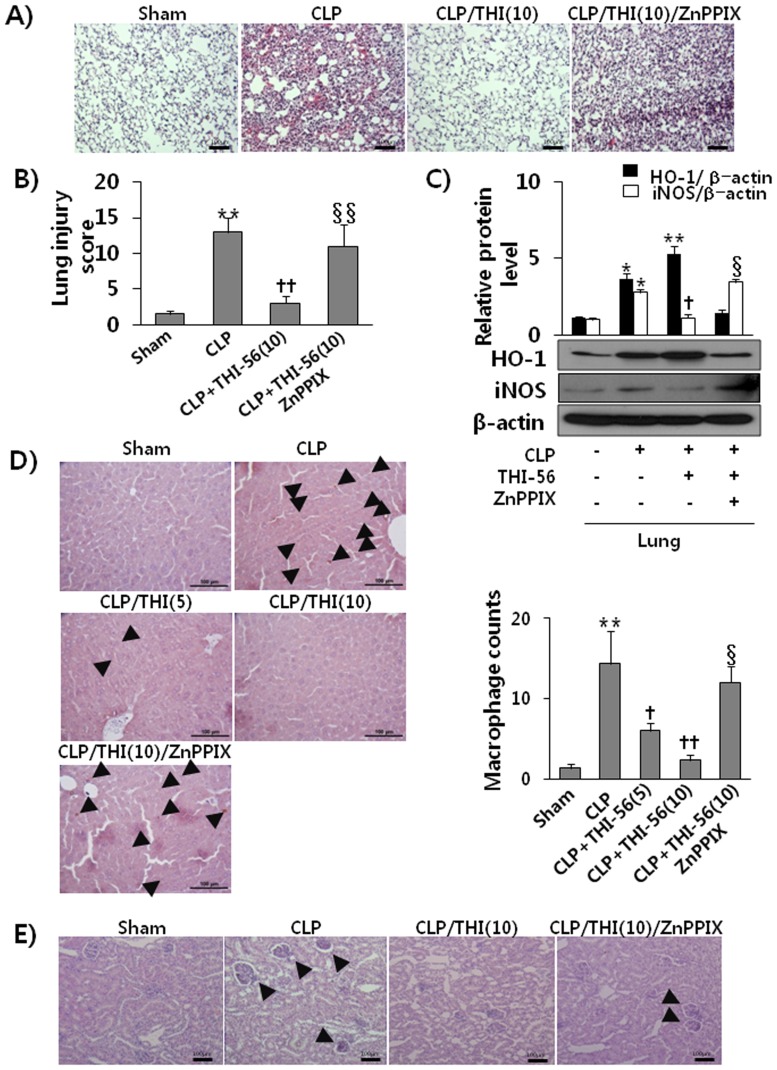
HO-1-dependent organ protective effect of THI-56 in CLP mice. BALB/c mice were treated with THI-56 (10 mg/kg, i.p., n = 5), THI-56 (10 mg/kg, i.p)+ZnPPIX (5 mg/kg, i.p., n = 5) or saline (i.p., n = 3) 2 h prior to CLP. After 24 h, the lungs, liver, and kidneys of each mouse were excised and processed as described in the Methods. The lung and kidney tissues were H&E-stained (A) and measured lung injury score as described in the Methods (B). Western blot analysis of iNOS and HO-1 protein expression in lung tissues was performed for the lungs of each group (C). The infiltration of macrophages in the livers of each group was assessed by staining with a macrophage marker (F4/80) (D). As marked by arrow, the histology of kidney demonstrated that CLP caused tubular injury, most prominent in the cortex, which was significantly attenuated by THI-56 in a ZnPPIX-sensitive manner (E). The data are presented as the means ± SEM. One-way analysis of variance followed by the Newman-Keuls test was used to compare the means of multiple groups (significant compared with the control, *P < 0.05; significant compared with CLP,^ †^ *P* < 0.05, significant compared with THI-56+CLP, ^§^ *P*<0.05).

Organ damage is one of leading causes of deaths in patients with sepsis. Thus, we investigated whether the protection against lung injury by THI-56 in CLP-induced septic mice was related to HO-1 activity. As shown in Fig. 6A and B, histopathological changes and lung injury were evident in lung and kidney tissues from mice with CLP-induced sepsis. It is clear that THI-56 reduced lung injury by reducing iNOS expression and upregulating HO-1 expression in lung tissues (Fig. 6C). In addition, the infiltration of macrophages into the liver also declined by THI-56 in a concentration-dependent and ZnPPIX-sensitive manner (Fig. 6D). Histopathological changes of kidney injury were clearly shown in CLP mice, whereas THI-56 reduced this injury in a ZnPPIX-sensitive manner (Fig. 6E). The data in Table 1 show that CLP increased levels of ALT, AST, BUN, and creatinine and that the administration of THI-56 significantly reduced the levels of markers of liver and kidney damage in a concentration-dependent manner.

**Table 1:** HO-1 dependence of protection of THI-56 against organ injury in CLP-induced septic mice.

Treatment	ALT (U/L)	AST (U/L)	BUN (U/L)	Creatinine (mg/dL)
Sham	73.3±23.8	320.1±100.9	18.5±0.4	0.38±0.35
CLP	350.9±145.6*	945.5±204.2**	37.1±2.3**	1.66±0.02*
CLP+THI56 (5 mg/kg)	298.6±106.7	803.2±309.7	23.8±3.2**^#^**	1.35±0.04**^#^**
CLP+THI56 (10 mg/kg)	274.1±7.4**^#^**	855.5±148.7	21.3±0.7**^##^**	0.83±0.14**^##^**
CLP+THI56 (5 mg/kg)+ZnPP (10 mg/kg)	369.0±16.5	865.1±150.9	37.6±3.4**^$^**	1.87±0.50**^$^**
CLP+THI56 (10 mg/kg)+ZnPP (10 mg/kg)	426.0±48.0**^†^**	1045.5±114.5	34.3±6.3**^†^**	1.21±0.41**^†^**

Mice were intraperitoneally administered 5 mg/kg or 10 mg/kg THI56 2 h prior to CLP. Twenty four h after CLP, blood was withdrawn under anesthesia by heart puncture from each group of animals. The results are presented as mean ± SEM (n  = 3–6). Significantly different (*P<0.05, **P<0.01) from sham. Significantly different (^#^ *P*<0.05, ^##^P<0.01) from CLP. Significantly different (^$^ *P*<0.05) from CLP+THI56 (5 mg/kg). Significantly different (**^†^** *P*<0.05) from CLP+THI56 (10 mg/kg).

## Discussion

The proinflammatory mediators expressed during sepsis can be roughly divided into two groups. The first group is the early proinflammatory mediators, such as TNF-α and IL-1β, which are induced within hours after the induction of sepsis. Initial experimental observations have suggested that the inhibition of these cytokines with neutralizing antibodies is beneficial in the treatment of sepsis [31]. However, clinical trials and more elegant animal studies have shown that the neutralization of early cytokines does not protect against sepsis [32]. Currently, it is suggested that early cytokines may be even protective against infection [32]. The second group of proinflammatory mediators is late-phase cytokines such as HMGB1, which are induced within 16–24 h after the initiation of sepsis. One interesting point is that increases in the late-phase cytokine HMGB1 are tightly associated with increased mortality in animal models of sepsis [7], whereas the administration of neutralizing antibodies to HMGB1 protects animals from death in the LPS and CLP models [7]–[8]. It has been proposed that the attenuation of the circulating levels of HMGB1 has a potential therapeutic uses for the treatment of sepsis. We showed that THI-56 significantly inhibits HMGB1 release, both *in vitro* and in *in vivo* sepsis models, indicating that THI-56 is a potential candidate for the treatment of sepsis.

HMGB1 is predominantly localized in the nucleus in resting macrophages. However, HMGB1 is translocated from the nucleus to the cytosol and subsequently to the extracellular milieu in response to different stimuli [5]. It has been suggested that several signaling proteins, such as C-jun N-terminal kinase (JNK), classical protein kinase C proteins (PKCs) and calcium/calmodulin-dependent protein kinase (CaMK) IV, are involved in HMGB1 shuttling in response to LPS stimulation in macrophages [33]–[35]. Moreover, it has also been claimed that the IFN-β/Janus kinase-2 (JAK-2)/signal transduction and activator of transcription-1 (STAT-1) signaling cascade is responsible for HMGB1 release in LPS-activated macrophages [36]. Interestingly, in our previous study, we showed that another THI alkaloid (CKD712) differentially regulates iNOS and COX-2 in LPS-treated cells through the negative regulation of the JAK-2/STAT-1 pathway [26]. It is possible that THI-56 can also interfere with this cascade. However, further study is needed to prove or disprove this hypothesis. In this study, we demonstrated that the inhibitory effect of THI-56 on HMGB1 release is mediated through HO-1 induction. These data strongly support our previous observations that the pharmacological or genetic upregulation of HO-1 greatly inhibits LPS-induced HMGB1 release both *in vitro* and *in vivo* [19]. In addition, the HMGB1 level is higher in HO-1^−/−^ mice than in HO-1^+/+^ mice when animals are subjected to either LPS injection or CLP-induced polymicrobial sepsis, indicating that HO-1 negatively regulates HMGB1 under septic conditions [18]–[19]. The present results indicate that the specific knockdown of the HO-1 gene using HO-1 siRNA greatly reversed the inhibitory effect of THI-56 on iNOS expression and HMGB1 release in LPS-stimulated macrophages. Thus, it might be important to provide evidence that the anti-inflammatory effect of THI-56 *in vivo* is mediated through HO-1. We therefore used the CLP-induced mouse model of sepsis. It is clear that the anti-inflammatory effects of THI-56 *in vivo* were dependent on HO-1 activity because the protective effect against organ damage was reversed by the administration of an HO-1 inhibitor. Another important finding is that the blood HMGB1 levels were significantly reduced by THI-56 in CLP-mice, and this effect was also significantly reversed by an HO-1 inhibitor, ZnPPIX, providing further evidence that HO-1 activity negatively regulates HMGB1 under systemic inflammatory conditions. Although the prevention of some of the complications of sepsis is important, whether THI-56 is therapeutically effective when administered after CLP is more clinically relevant. We found that even when THI-56 was administered 12 h after CLP, it still reduced the circulating HMGB1 levels, suggesting that this drug can potentially be beneficial in treating sepsis. THI-56 induced the translocation of Nrf-2 from the cytosol to the nucleus in a concentration-dependent manner and the deletion of Nrf-2 significantly diminished HO-1 expression in RAW 264.7 cells; therefore, the anti-inflammatory effects of THI-56 are mediated through the up-regulation of HO-1 expression via Nrf2 activation.

In summary, this study demonstrated that THI-56 improves survival in lethal endotoxemia and CLP-induced sepsis models. THI-56 significantly inhibited LPS-induced iNOS/NO and HMGB1 release in macrophages and reduced organ damage in CLP-induced septic mice. The beneficial effects of THI-56 were dependent on its ability to induce HO-1 expression through the activation of the Nrf-2 transcription factor. Because increased HMGB1 expression is tightly associated with increased mortality in animal models of sepsis, it should be noted that the administration of THI-56 even 12 h after CLP decreased the circulating HMGB1 levels in CLP mice. Therefore, THI-56 may be useful in the treatment of sepsis.
